# Magnetisation transfer ratio combined with magnetic resonance neurography is feasible in the proximal lumbar plexus using healthy volunteers at 3T

**DOI:** 10.1038/s41598-020-71570-1

**Published:** 2020-09-03

**Authors:** Marios C. Yiannakas, Torben Schneider, Masami Yoneyama, Innocent Aforlabi-Logoh, Ferran Prados, Olga Ciccarelli, Claudia A. M. Wheeler-Kingshott

**Affiliations:** 1 NMR Research Unit, Queen Square MS Centre, Department of Neuroinflammation, UCL Queen Square Institute of Neurology, Faculty of Brain Sciences, University College London, Queen Square House, Queen Square, London, WC1N 3BG UK; 2 Philips Healthcare, Guildford, Surrey UK; 3 Philips Japan, Tokyo, Japan; 4 Centre for Medical Image Computing, Medical Physics and Biomedical Engineering Department, University College London, London, UK; 5 e-Health Centre, Universitat Oberta de Catalunya, Barcelona, Spain; 6 Brain MRI 3T Research Centre, IRCCS Mondino Foundation, Pavia, Italy; 7 Department of Brain and Behavioural Sciences, University of Pavia, Pavia, Italy

**Keywords:** Neurology, Peripheral nervous system

## Abstract

Magnetic resonance neurography (MRN) has been used extensively to study pathological conditions affecting the peripheral nervous system (PNS). However, tissue damage is assessed qualitatively with little information regarding the underlying pathophysiological processes involved. Magnetisation transfer ratio (MTR) is a quantitative magnetic resonance imaging method which is sensitive to tissue macromolecular content and may therefore have an important role in the study of pathologies affecting the PNS. This study explored the feasibility of obtaining reliable MTR measurements in the proximal lumbar plexus of healthy volunteers using MRN to identify and segment each lumbar segment (L2–L5) and regions (preganglionic, ganglionic and postganglionic). Reproducibility of the MTR measurements and of the segmentation method were assessed from repeated measurements (scan-rescan), and from the reanalysis of images (intra- and inter-rater assessment), by calculating the coefficient of variation (COV). In all segments combined (L2–L5), mean (± SD) MTR was 30.5 (± 2.4). Scan-rescan, intra- and inter-rater COV values were 3.2%, 4.4% and 5.3%, respectively. One-way analysis of variance revealed a statistically significant difference in MTR between the preganglionic and postganglionic regions in all lumbar segments. This pilot study in healthy volunteers demonstrates the feasibility of obtaining reliable MTR measurements in the proximal lumbar plexus, opening up the possibility of studying a broad spectrum of neurological conditions in vivo.

## Introduction

Magnetic resonance neurography (MRN) has been used extensively in recent years to study inflammatory, neoplastic, metabolic and traumatic pathologic conditions affecting the peripheral nervous system (PNS)^1–3^. Typically, high-resolution fat-suppressed three-dimensional (3D) T2-weighted (T2w) turbo spin-echo (TSE) sequences are used, which offer excellent tissue contrast and allow reformatting in multiple planes for easier visualisation of the peripheral nerves^4–7^. However, tissue damage is assessed qualitatively by observing changes in T2w signal intensity and/or cross-sectional area of the peripheral nerves, and such observations provide little information regarding the underlying pathophysiological mechanisms involved in each case; understanding these mechanisms not only could aid early diagnosis but could provide avenues to explore new therapeutic interventions.

Recently, investigators have employed quantitative magnetic resonance imaging (MRI) methods to study the PNS, such as relaxometry for estimating T2 relaxation time and proton density, with reported applications in healthy volunteers^8^, chronic inflammatory demyelinating polyradiculoneuropathy^9^, multiple sclerosis^10^ and diabetic and amyloidotic polyneuropathy^11,12^. These studies have demonstrated that the increased T2w signal intensity typically observed in MRN investigations may not always reflect an increase in T2 relaxation time but also alteration in proton density, supporting the notion that macromolecular structures may play an important role^9–12^.

Magnetisation transfer (MT) imaging is a quantitative MRI method, which has been used in the central nervous system (CNS) to study the interaction between ‘restricted protons’ (i.e. protons bound to macromolecules) and ‘free protons’^13,14^, and has been shown to correlate highly with tissue myelin content in the brain^15^ and spinal cord^16^. Recent studies using healthy volunteers sought to investigate the feasibility of measuring magnetisation transfer ratio (MTR) in the PNS, and in particular to determine whether nerves from different anatomical locations such as the foot and the wrist have different MR characteristics, demonstrating vast differences in MTR between them^17^. In another study, the MTR of lower extremity nerves showed no proximal-to-distal gradient in young healthy volunteers but was found to decrease with age^18^. Furthermore, in the first reported clinical application of MTR in patients with Charcot-Marie-Tooth disease, it was demonstrated that MTR was significantly decreased in the proximal sciatic nerve of patients as compared to controls suggesting that MTR measurements may be a viable biomarker of proximal nerve pathology^19^. The ability to obtain reliable MTR measurements in the peripheral nerves of the lower extremities as well as other anatomical locations such as the lumbar plexus for example, which is known to be affected by a plethora of neurological conditions^20,21^, can have profound clinical implications both in terms of diagnosis and approach to treatment.

In this pilot study, the feasibility of obtaining MTR measurements in the proximal lumbar plexus was explored in healthy volunteers using the detailed structural information obtainable with an established MRN method^6,7^; this has the potential of providing a reliable and clinically viable index sensitive to macromolecular content at this level, opening up the possibilities to study a broad spectrum of neurological conditions in vivo.

## Materials and methods

### Study participants

Ten healthy volunteers [mean ± standard deviation (SD) age 36.4 (± 9.5) years, range 29–61, 5 female] were recruited, with five of them returning for a repeat scan. The study was conducted in line with the International Conference on Harmonisation Good Clinical Practice (ICH GCP) and was approved by the London – Harrow Research Ethics Committee. Written informed consent was obtained from all study participants prior to their scans.

### MR imaging

**Figure 1:**
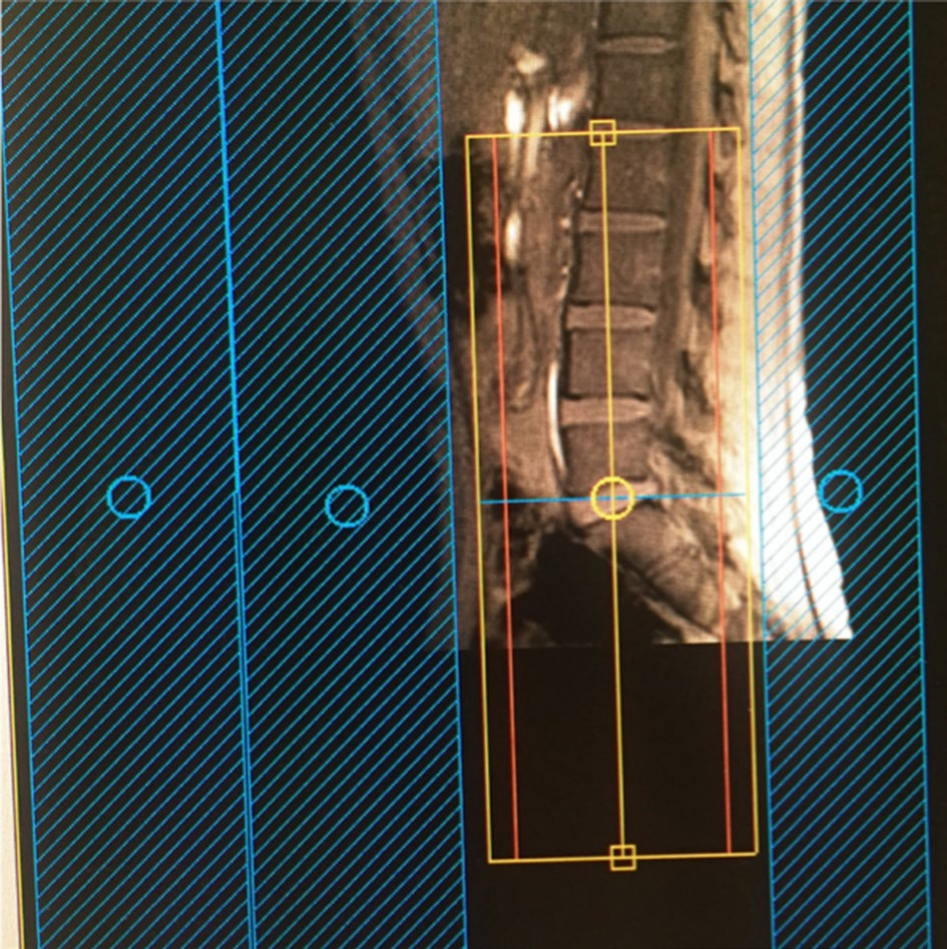
Imaging volume (yellow) and shim box (red) prescription using the 3D ‘nerve-SHeath signal increased with INKed rest-tissue RARE Imaging’ (SHINKEI) sequence, starting from the superior margin of the L2 vertebral body extending caudally towards the sacral segments, to ensure coverage of L2–L5 segments in all subjects. Positioning of anterior and posterior saturation slabs is also shown (blue).

**Figure 2:**
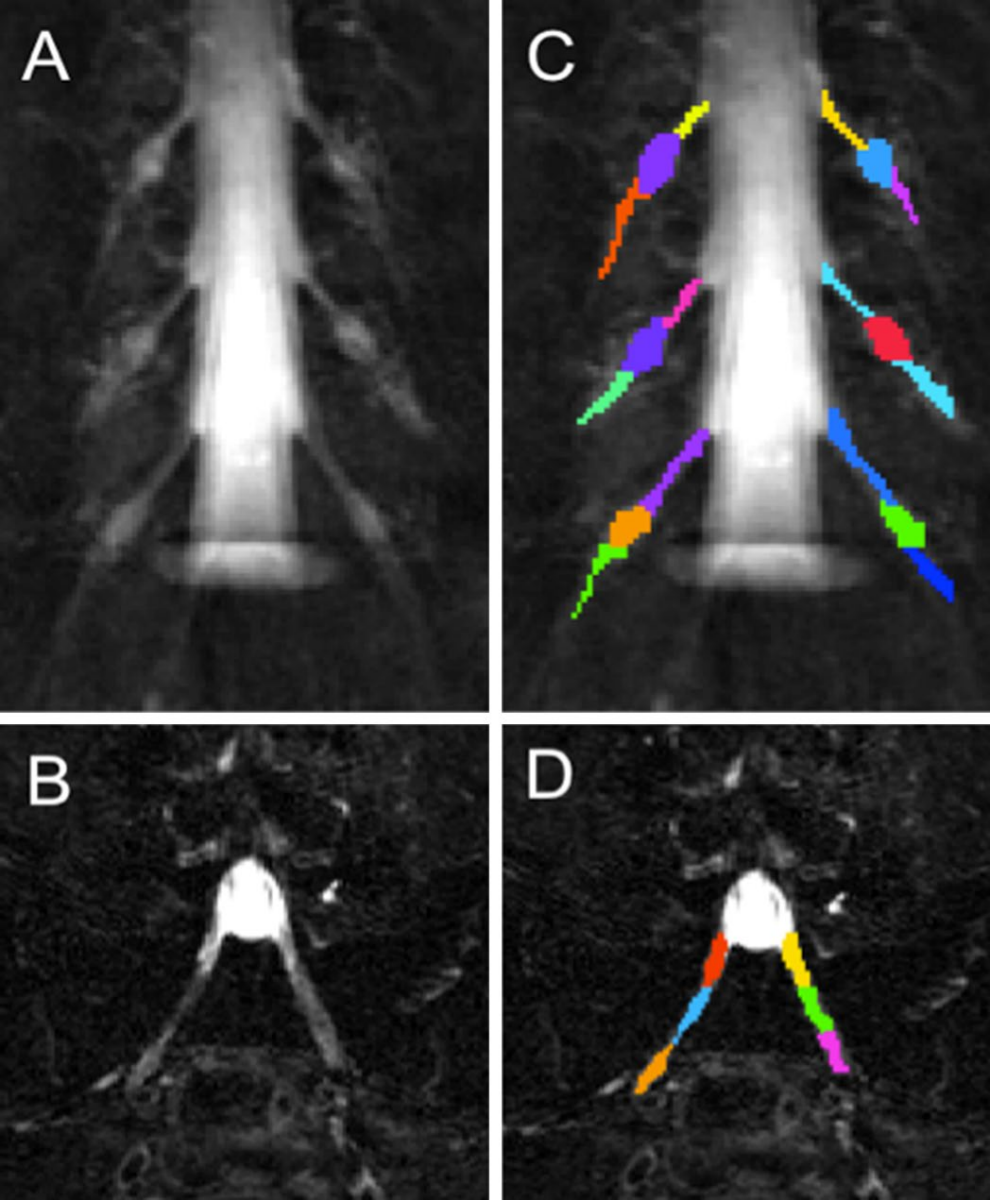
Example images obtained using the ‘nerve-SHeath signal increased with INKed rest-tissue RARE Imaging’ (SHINKEI) sequence showing (in the superior-inferior direction) (**A**) the L2–L4 segments and (**B**) the L5 segment; (**C**) annotated binary masks were drawn manually for each segment (L2, L3, L4) and (**D**) L5, to distinguish between the preganglionic, ganglionic and postganglionic regions.

Using a Philips Achieva 3 T and the product 15-channel SENSE spine coil, the lumbosacral spine was imaged coronally using the 3D ‘nerve-SHeath signal increased with INKed rest-tissue RARE Imaging’ (SHINKEI) sequence^6,7^, as follows: TR = 2,500 ms; TE = 192 ms, FOV = 180 × 180 mm^2^, voxel size = 1 × 1 × 1 mm^3^, number of averages = 1, TSE factor = 100, iMSDE duration = 50 ms, 81 slices, scanning time of 10:20 min. The imaging volume was positioned at the superior margin of the L2 vertebral body with the volume extending caudally towards the sacral segments, to ensure coverage of L2-L5 segments in all subjects (Fig. 1); an example image is shown in Fig. 2A,B. MTR imaging was performed using identical scan geometry to the SHINKEI acquisition as follows: 3D FFE dual-echo ‘stack of stars’ (TR / TE1 / TE2 = 36 / 1.56 / 2.7 ms; flip angle α = 10°), with and without Sinc–Gaussian shaped MT saturation pulses with nominal α = 360°; offset frequency = 1 kHz, and duration 16 ms; number of slices = 81; FOV = 180 × 180 mm^2^; voxel size = 1 × 1 × 1 mm^3^; scanning time of 10:45 min.

### Image analysis

**Figure 3:**
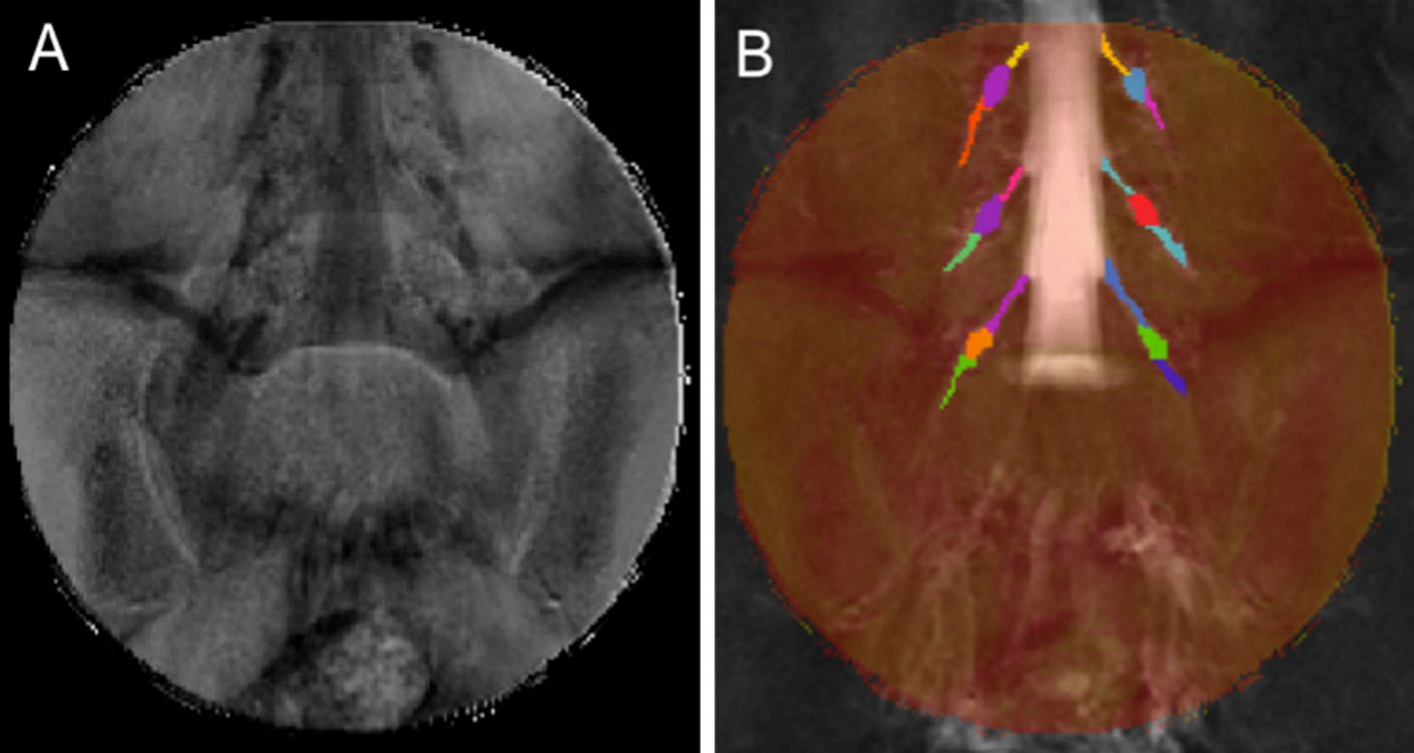
(**A**) Example of a magnetisation transfer ratio (MTR) map calculated in one healthy volunteer; (**B**) each MTR map was registered to the respective ‘nerve-SHeath signal increased with INKed rest-tissue RARE Imaging’ (SHINKEI) volume with annotated binary masks subsequently applied to obtain MTR values for each lumbar segment (only L2, L3, L4 shown here) as well as the preganglionic, ganglionic and postganglionic regions.

Image segmentation was performed manually in FSLview (https://www.fmrib.ox.ac.uk/fsl/) using the SHINKEI images. For each lumbar segment (L2-L5), annotated binary masks were used to distinguish between preganglionic, ganglionic and postganglionic regions of similar volume (Fig. 2C,D). All MTR volumes were subsequently co-registered to their respective SHINKEI volumes using affine registration with NiftyReg^22^. Binary annotated masks were resampled applying the affine transformation and using nearest-neighbor interpolation in order to obtain segment- and region-specific MTR values (Fig. 3).

### Reproducibility assessment

The reproducibility of the MTR measurements was assessed by performing a ‘scan-rescan’ test in five out of ten healthy volunteers with a minimum of 7 days (and a maximum of 14 days) in between the first and the second scan. One rater with 12 years experience in neuroimaging research (MCY) performed all the data analysis. In addition, to demonstrate the intra-rater reproducibility of the segmentation method, the same rater analysed all the data from the five volunteers’ first visit twice; the analysis was performed on separate occasions with a minimum of 2 weeks between each analysis and with the images presented to the rater in random order. Inter-rater reproducibility was assessed with a second rater (IA-L) with 3 years of experience in neuroimaging research, who also analysed the data from the 5 volunteers’ first visit. The two raters worked independently using predefined guidelines and were unaware of the results of each other.

### Statistical analysis

Statistical analysis was performed using SPSS 24.0 (SPSS, Chicago, Ill., USA). Firstly, differences in MTR values between the left and right lumbar segments were investigated using paired* t* tests. For the assessment of scan-rescan, intra-rater and inter-rater reproducibility of the MTR measurements, the coefficient of variation (COV) and intraclass correlation coefficient (ICC) were calculated. The COV was calculated using the mean and standard deviation from the repeated measurements and the equation COV = [SD/mean] × 100%. The ICC for inter-rater reproducibility was calculated using a two-way random model, whereas for intra-rater and scan-rescan reproducibility, this was calculated using a two-way mixed model. In all cases, the ICC was interpreted as showing ‘poor agreement’ (value less than 0.4), ‘good agreement’ (value of 0.4–0.75) or ‘excellent agreement’ (value of 0.75 or greater). Furthermore, to assess intra- and inter-rater reproducibility of the manual segmentation method the Dice similarity coefficient (DSC) was obtained^23^. The DSC is a measurement of spatial overlap between two sets and represents the size of the union of two sets divided by the average size of the two sets; a value of 0 indicates no overlap while a value of 1 indicates perfect agreement. Differences in MTR values between preganglionic, ganglionic and postganglionic regions in each individual lumbar segment were investigated using one-way analysis of variance (ANOVA) with post hoc comparisons using the Tukey test.

## Results

Paired* t* tests in ten healthy volunteers revealed no statistically significant differences in MTR values between left and right lumbar segments. As such, all subsequent analyses for each lumbar segment were performed using average values i.e. (left + right)/2.

The mean (± SD) MTR of all lumbar segments combined (L2-L5) in the 10 healthy volunteers (averaged over all regions i.e. pre-, post-ganglionic and side i.e. L/R) was 30.5 (± 2.4). Using five healthy volunteers, scan-rescan, intra- and inter-rater COV values for all lumbar segments combined (L2–5) were 3.2%, 4.4% and 5.3%, respectively. The scan-rescan, intra- and inter-rater ICC values for all lumbar segments combined (L2–5) were 0.76, 0.79 and 0.65, respectively. Table 1 reports the mean (± SD) MTR values (N = 10), COV values (N = 5) and ICC values (N = 5) obtained in each lumbar segment separately.

**Table 1:** Magnetisation transfer ratio (MTR) values measured in each lumbar segment (averaged over all regions i.e. pre-, post-ganglionic and side i.e. L/R) (N = 10) and scan-rescan, intra-rater and inter-rater reproducibility results (N = 5).

Measurement	Mean MTR (a.u.)	SD	Scan-rescan (%COV)	Intra-rater (%COV)	Inter-rater (%COV)	Scan-rescan (ICC)	Intra-rater (ICC)	Inter-rater (ICC)
L2	29.4	4.5	6.8	4.8	8.0	0.86	0.90	0.79
L3	31.0	2.5	3.6	4.3	4.9	0.56	0.78	0.59
L4	31.1	3.3	6.0	5.4	6.1	0.35	0.84	0.74
L5	30.6	3.5	4.9	5.5	3.1	0.79	0.89	0.96
All (L2-L5)	30.5	2.4	3.2	4.4	5.3	0.76	0.79	0.65

*MTR* magnetisation transfer ratio, *SD* standard deviation, *COV* coefficient of variation, *ICC* Intraclass correlation coefficient, *a.u* arbitrary units.

**Figure 4:**
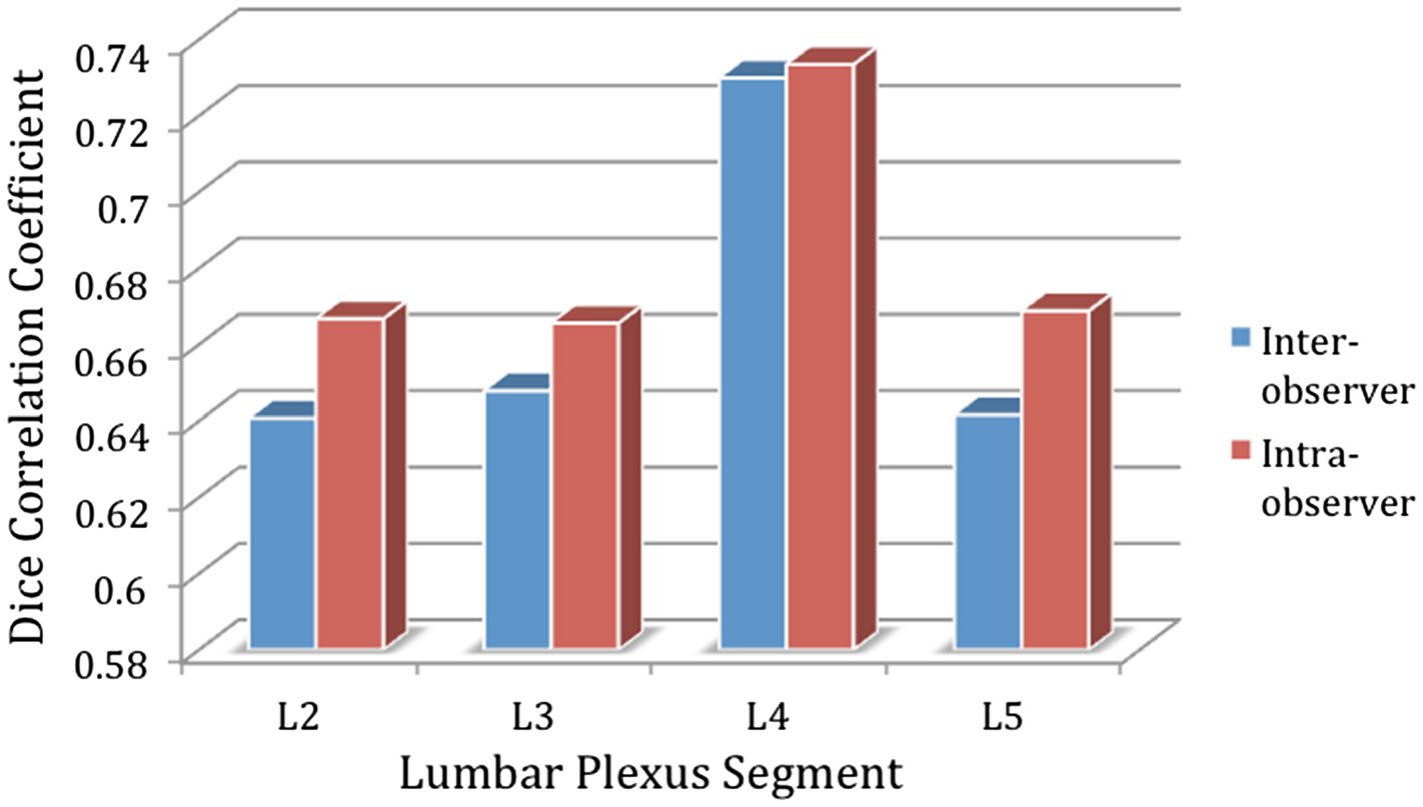
Mean Dice similarity coefficient (DSC) of the manually segmented binary masks for each lumbar segment in five healthy volunteers obtained from a single rater (intra-rater) and two raters (inter-rater).

In five healthy volunteers, mean (± SD) DSC of the manually segmented binary masks for all lumbar segments combined (L2-L5) for a single rater (intra-rater) was 0.72 (± 0.09) and for two raters (inter-rater) was 0.67 (± 0.06). Figure 4 shows the intra- and inter-rater DSC results for each lumbar segment separately.

Using one-way analysis of variance (ANOVA) in ten healthy volunteers, a statistically significant difference in MTR was identified between regions (preganglionic, ganglionic, postganglionic) in every lumbar segment. Using the Tukey post hoc test it was shown that, in all segments, there was a statistically significant difference between the preganglionic and postganglionic regions, with a further significant difference between preganglionic and ganglionic regions at L5 segment and in all segments combined (L2-L5). Table 2 shows MTR values measured per region in each lumbar segment and results of the ANOVA with all post hoc comparisons using the Tukey test.

**Table 2:** Magnetisation transfer ratio (MTR) values measured per region in each lumbar segment (average left/right) (N = 10) and results of the one-way analysis of variance (ANOVA) with post hoc comparisons using the Tukey test (*p* value).

Segment	Region	Mean MTR (a.u.)	SD	*p* value
	Preganglionic^a^	24.8	7.3	L2^a,b^ = 0.11
L2	Ganglionic^b^	29.9	3.9	L2^a,c^ = 0.004*
	Postganglionic^c^	33.4	4.3	L2^b,c^ = 0.33
	Preganglionic^a^	29.3	2.6	L3^a,b^ = 0.20
L3	Ganglionic^b^	31.2	2.6	L3^a,c^ = 0.02*
	Postganglionic^c^	32.5	2.2	L3^b,c^ = 0.5
	Preganglionic^a^	29.1	3.0	L4^a,b^ = 0.37
L4	Ganglionic^b^	31.0	3.0	L4^a,c^ = 0.04*
	Postganglionic^c^	32.6	3.0	L4^b,c^ = 0.45
	Preganglionic^a^	26.9	6.0	L5^a,b^ = 0.04*
L5	Ganglionic^b^	31.8	3.5	L5^a,c^ = 0.01*
	Postganglionic^c^	32.9	2.7	L5^b,c^ = 0.84
	Preganglionic^a^	27.7	2.8	All^a,b^ = 0.02*
All	Ganglionic^b^	31.0	2.4	All^a,c^ = 0.001*
	Postganglionic^c^	32.9	2.2	All^b,c^ = 0.23

*MTR* magnetisation transfer ratio, *SD* standard deviation, *a.u* arbitrary units.

* indicates statistical significance (*p* value <0.05).

^a, b, c^ indicate the ANOVA post hoc pairwise results.

## Discussion

In this pilot study, we have demonstrated the feasibility of obtaining reliable MTR measurements in the proximal lumbar plexus in healthy volunteers using a 3 T MRI system and commercially available software and hardware; the acquisition and analysis protocol presented in this work maybe easily translated to the clinical settings to study a large number of pathological conditions affecting the lumbar plexus^20,21^. The rationale behind this study was the lack of quantitative MRI protocols that can be used to obtain more specific information about the underlying pathophysiological processes involved in common pathologies affecting the lumbar plexus. Given the large spectrum of peripheral demyelinating diseases^24^, the use of a more biophysically meaningful in vivo MRI biomarker such as MTR, which has been shown to be directly influenced by the amount of myelin in neural tissue^15,16^, would be appropriate not only for aiding diagnosis but potentially guiding appropriate therapeutic interventions.

In order to understand the origin of the MTR contrast in the PNS, it is prudent to consider the differences in tissue structure and composition of the nerves in the PNS as compared to the CNS. In the PNS, the outer surface of the peripheral nerve is a layer of fibrous connective tissue, the epineurium. Within the nerve, axons are bundled into fascicles, with each fascicle surrounded by another layer of fibrous tissue, the perineurium. Finally, individual myelinated (or unmyelinated) axons are surrounded by loose connective tissue, the endoneurium. Thus, in the context of MTR measurements, the ‘restricted protons’ are represented by collagen tissue, myelin and the proteins contained in the axons and Schwann cells^18^; however, the relative contribution of each tissue type to the measureable MTR remains unknown.

There are only few studies in the literature reporting MTR measurements in the PNS of humans in vivo. One study sought to determine in healthy volunteers whether nerves from different anatomical locations such as the foot and the wrist have different MR characteristics, demonstrating vast differences in MTR between them^17^, and in another study the MTR of lower extremity nerves showed no proximal-to-distal gradient in young healthy volunteers, but was found to decrease with age^18^. In the first reported clinical application of MTR in patients with CMT diseases^19^, mean MTR values in the sciatic nerve were shown to reduce in CMT type 1A and CMT type 2A by 10% and 15%, respectively. Considering the mean MTR (SD) values obtained in our own study in healthy controls, to detect a 10% or 15% change with 80% power at 5% significance in the proximal lumbar plexus, future studies should recruit a minimum of 20 subjects (10 patients and 10 controls) or 10 subjects (5 patients and 5 controls), respectively.

Identifying the extent of variability in normative MTR values at different anatomical locations can be invaluable in determining the potential clinical utility of these measurements. However, MTR is a semi-quantitative parameter and its value depends on many factors, such as the MRI pulse sequence implemented, the saturation pulse type and amplitude, B1 field inhomogeneities, to name a few; as such, a comparison of MTR between sites may not always be possible^25^, although normative MTR values obtained under the same experimental conditions and at single sites can be especially useful.

In this study, we found that there was no statistically significant difference in MTR between right and left lumbar segments. However, a statistically significant difference was found between preganglionic and postganglionic regions. In particular, MTR values were higher in the postganglionic regions as compared to the preganglionic regions in all lumbar segments. One possible biological explanation maybe the difference in tissue composition between these two regions, in particular the presence of collagen tissue in the postganglionic regions, which maybe driving the higher MTR values. The MTR values within the ganglionic regions were of intermediate value, possibly because these regions predominantly contain cell bodies of neurons in afferent spinal nerves, hence of different tissue composition to both preganglionic and ganglionic regions. Interestingly, the MTR values in this study display an increasing value moving from preganglionic to postganglionic regions, whereas in a recent similar study measuring T2 relaxation time, T2 was the longest in the ganglionic regions, shorter in the preganglionic regions and shortest in the postganglionic regions^8^, suggesting that MTR and T2 most probably reflect changes in different proton pools.

The reproducibility of MTR measurements in this study demonstrated a mean scan-rescan, intra- and inter-rater % coefficient of variation for all segments combined (L2-L5) of 3.2%, 4.4% and 5.3%, respectively. While there are no similar studies in the literature with which to directly compare these figures, these results are very encouraging as they are comparable to those previously obtained in the lower spinal cord grey- and white-matter^26^. Furthermore, since much smaller regions were evaluated in this study, the results are possibly reflecting more errors related to technical factors and/or partial volume averaging. This consideration is in part supported by the calculated mean (± SD) DSC of the manually segmented binary masks for all lumbar segments combined (L2-L5) for a single rater (intra-rater) and two raters (inter-rater), which were found to be 0.72 (± 0.09) and 0.67 (± 0.06), respectively; these results are considerably lower than what is typically measured in the lower cord cross-sectional area (intra-rater, 0.97 ± 0.01; inter-rater, 0.97 ± 0.01) or the lower cord grey-matter cross-sectional area (intra-rater, 0.89 ± 0.01; inter-rater, 0.88 ± 0.01)^27^. Future work must therefore be directed at reducing operator input, for example through the use of lumbar plexus templates, or fully-automated image analysis methods depending on the specific application.

In this pilot study we have demonstrated the feasibility of obtaining reliable MTR measurements within the proximal lumbar plexus in healthy volunteers using a 3 T MRI system and commercially available software and hardware. The acquisition and analysis protocol presented can be easily implemented in the clinical setting to study a broad spectrum of pathological conditions affecting the lumbar plexus.
